# Supplementary material for: The interactome of intact mitochondria by cross-linking mass spectrometry provides evidence for coexisting respiratory supercomplexes
Source: Mol Cell Proteomics. 2017 Dec 8;17(2):216–32. doi: 10.1074/mcp.RA117.000470 (PMC5795388; doi:10.1074/mcp.RA117.000470)
Supplement: Supplemental Data [file supp_RA117.000470_133922_0_supp_23978_fzffwf.zip › spectra_annotation/mito_DR_spectra_annotation/450-1-8-1-11-1.pdf]

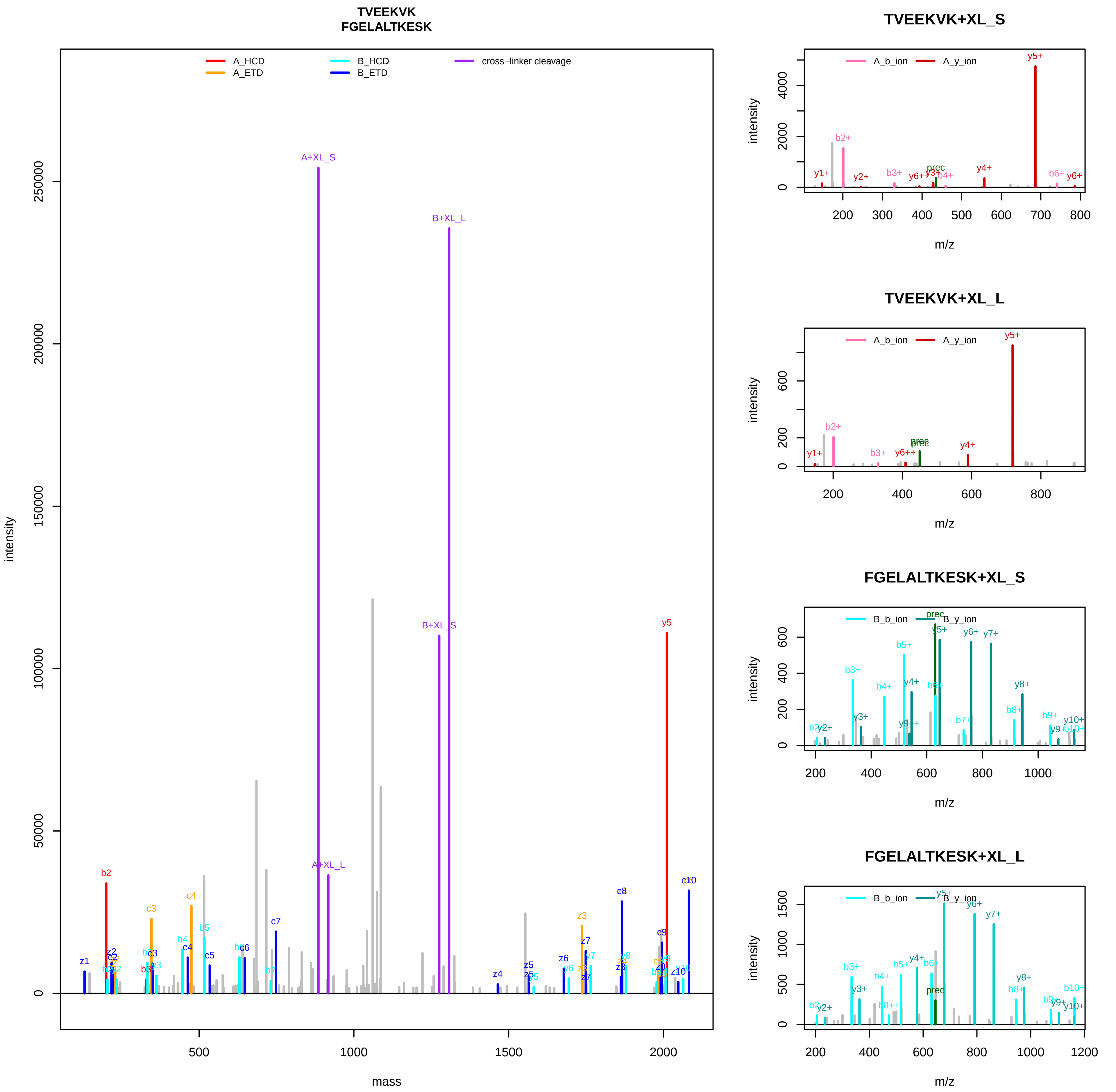
TVEEKVK
FGELALTKESK
A_HCD
A_ETD
B_HCD
B_ETD
cross−linker cleavage
A+XL_S
B+XL_L
B+XL_S
A+XL_L
y5
b2
c3
c4
c5
c6
c7
c8
c9
c10
z1
z2
z3
z4
z5
z6
z7
z8
z9
z10
b4
b5
y7
y8
y10
intensity
mass
0
50000
100000
150000
200000
250000
500
1000
1500
2000
TVEEKVK+XL_S
A_b_ion
A_y_ion
y1+
b2+
y2+
b3+
y6++
y3+
prec
b4+
y4+
y5+
b6+
y6+
intensity
m/z
0
2000
4000
200
300
400
500
600
700
800
TVEEKVK+XL_L
A_b_ion
A_y_ion
y1+
b2+
b3+
y6++
prec
y4+
y5+
intensity
m/z
0
200
600
200
400
600
800
FGELALTKESK+XL_S
B_b_ion
B_y_ion
prec
b3+
y3+
b4+
y4+
b5+
y5+
y6+
y7+
b7+
b8+
y8+
b9+
y10+
intensity
m/z
0
200
400
600
200
400
600
800
1000
FGELALTKESK+XL_L
B_b_ion
B_y_ion
b3+
y3+
b4+
b5+
y4+
b6+
y5+
y6+
y7+
prec
b8+
y8+
b9+
y9+
b10+
y10+
intensity
m/z
0
500
1000
1500
200
400
600
800
1000
1200
